# Characterization of a new highly sensitive immunometric assay for thyroglobulin with reduced interference from autoantibodies

**DOI:** 10.1007/s13277-015-4597-2

**Published:** 2015-12-22

**Authors:** Marianne Nordlund Broughton, Ragnhild Nome, Ingvill Sandven, Elisabeth Paus, Trine Bjøro

**Affiliations:** 1 Department of Medical Biochemistry, Radiumhospitalet, Oslo University Hospital (OUH), Oslo, Norway; 2 Institute of Clinical Medicine, University of Oslo, Oslo, Norway

**Keywords:** Thyroid carcinoma, Thyroglobulin, Autoantibodies, Immunoassay, Time-resolved

## Abstract

Measurements of serum thyroglobulin (Tg) with sensitive immunoassays are of great importance for the management of patients with differentiated thyroid carcinomas. However, interference of circulating autoantibodies to Tg (hTgAb) hampers the usefulness of most assays. We have produced a panel of monoclonal antibodies (mAbs) selected to bind Tg in the presence of Tg autoantibodies and developed a sensitive immunoassay for Tg with minor interference by hTgAbs. The antibodies were characterized by cross-inhibition and immunoassay combination studies, as well as affinity estimation. The within-run and total imprecision of the assay were determined with 2664 samples in 60 separate runs. The most sensitive assay combination with superior protection against autoantibodies consisted of two solid phase mAbs and two tracer mAbs with distinct binding sites. The assay was linear and displayed a wide dynamic range up to 1342 μg/l with a functional sensitivity of 0.1 μg/l and a total imprecision of less than 10 %. There was good agreement between the new high sensitive immunofluorometric assay (IFMA) and two well-established Tg assays from Brahms Kryptor and Roche Diagnostics. Mean difference between the new IFMA and the Kryptor assay was 0.059 μg/l with a 95 % confidence interval of −0.032 to 0.151 μg/l, whereas the mean difference between the new IFMA and the Roche assay was −0.80 μg/l with a 95 % confidence interval of −1.24 to −0.35 μg/l.

**Electronic supplementary material:**

The online version of this article (doi:10.1007/s13277-015-4597-2) contains supplementary material, which is available to authorized users.

## Introduction

Thyroglobulin (Tg) is a 670-kDa glycoprotein produced specifically by the follicular cells of the thyroid gland. The Tg molecule consists of two identical subunits which undergo extensive posttranslational modification including glycolsylation, phosphorylation, sulfation, and iodination [1, 2]. Ultimately, thyroglobulin functions as a prohormone for the thyroid hormones thyroxine (T_4_) and triiodothyronine (T_3_), but it is also released from the thyroid gland and is detectable in sera of most normal individuals.

The incidence of thyroid cancer has increased by 50 % over the past 25 years [3, 4]. The most important clinical application of an immunoassay for thyroglobulin is in the follow-up of patients with differentiated thyroid cancer (DTC) which have undergone total thyroidectomy. DTCs are often diagnosed in the early decades of life, and recurrences can occur many years after the primary treatment, necessitating life-long monitoring with clinical examinations and serial serum Tg measurements. The reoccurrence of serum Tg after thyroidectomy and 131-iodine ablation strongly indicates metastatic disease, and small elevation in the 0.2 μg/l range can be of pathological significance. The treatment and follow-up of this patient group have also changed, and the American Thyroid Association guidelines suggest that Tg assays with functional sensitivity of ~0.1 μg/l may reduce the need to perform TSH-stimulated Tg measurements during the initial follow-up of some patients [5–7]. Thus, the sensitivity of the assay is very important for its clinical usefulness. Another challenge is assay interference by human autoantibodies against thyroglobulin (TgAb). In immunometric assays, these autoantibodies can cause an underestimation of the thyroglobulin concentration and, consequently, reduce the usefulness of Tg in the follow-up of these patients. Circulating autoantibodies to Tg are often positive in patients with autoimmune thyroid diseases, Hashimoto’s thyroiditis (HT), and Graves’ disease (GD), but TgAbs can also be detected in patients with thyroid carcinoma and in individuals with no apparent thyroid disease. It has been shown that Tg autoantibodies are detected in approximately 20 % of patients with thyroid cancer and in approximately 10 % of normal individuals [8]. To reveal TgAb interference in Tg assays, there are guidelines recommending reliable TgAb detection prior to Tg testing by immunoassays [8]. A negative TgAb test is used to verify the absence of TgAb interference, whereas a positive TgAb test indicates that the Tg concentration measured in the immunoassay may be unreliable and give a falsely low/undetectable serum Tg concentration that could mask disease. This could have serious consequences for the follow-up of patients with DTC which have undergone total thyroidectomy, and unfortunately, it has been reported that false-negative TgAb misclassification was 30–40 % using manufacturer-recommended TgAb cutoffs [9]. Thus, there is a clear need for a new, improved, and highly sensitive immunoassay for Tg with less interference by Tg autoantibodies.

Here we describe the production of a panel of monoclonal antibodies selected to bind Tg in the presence of human autoantibodies from cancer patients. We have further characterized the antibody specificities and tested suitable antibody pairs for construction of an immunofluorometric assay (IFMA) for Tg. The assay was finally compared to two well-established immunoassays for Tg (Brahms Kryptor and Roche Diagnostics).

## Materials and methods

### Production of monoclonal antibodies

Female BALB/c mice (6–8 weeks of age; Harlan Olac Ltd., Oxon, UK) were primed by subcutaneous injections with 25 μg Tg emulsified in Freund’s complete adjuvant (for antigen preparation see Supplemental data). Two booster immunizations of 30 μg of Tg (subcutaneous) in Freund’s incomplete adjuvant were given 2–3 and 4–5 months later. One month after the last boost immunization dose, four daily intraperitoneal boosts of 50–200 μg of Tg were given immediately before splenectomy [10]. Experimental animals were treated in accordance with institutional guidelines. Specific immune response was determined by incubating 100 μl diluted blood samples from immunized mice with 100 μl ^125^I-labelled Tg (approximately 50,000 cpm) in 0.05 M Tris–HCl buffer pH 7.8 containing 1 % bovine serum albumin (BSA). Incubation was performed at room temperature before free and bound antigen was separated using sheep anti-mouse antibodies (SAM) coupled to paramagnetic polymer particles (Dynabeads M280, Life Technologies, Oslo, Norway), by adding 100 μl of a 10 mg/ml suspension. The resulting hybridomas were screened for anti-Tg monoclonal antibodies in absence and presence of human anti-thyroglobulin antibodies (for primary and secondary screening procedures, see Supplemental data) [11]. After the second screening, hybridomas were subcloned and selected clones were chosen for further in vitro expansion. Antibodies were purified by Protein A-Sepharose 4B (GE Healthcare Life Sciences, Uppsala, Sweden) chromatography. Subtyping of mAbs was performed using the IsoStrip Mouse Monoclonal Antibody Isotyping Kit (Roche, Indianapolis, IN, USA).

### Measuring human autoantibodies to TgAb

Human TgAb was analyzed using a competitive assay (Brahms Kryptor, Henningsdorf, Germany) with a measuring range between 10 and 850 kU/l. When TgAb levels were above 850 kU/l, the samples were automatically diluted and the measuring range for automatically dilution was 10–20,000 kU/l.

### Radiolabelling of Tg

Thyroglobulin and antibodies were iodinated by the indirect iodogen method (Pierce, Rockford, IL, USA) with Na^125^I (Amersham Pharmacia Biotech, Little Chalfont, UK) at an equal molar ratio of protein to iodine. Iodinated protein was stored at −20 °C in 50 % ethylene glycol and 0.05 M Tris–HCl buffer pH 7.8 containing 1 g/l BSA.

### Conjugation of tracer antibodies with Eu^3+^-chelates

Purified antibodies were conjugated to a europium chelate using the DELFIA Eu^3+^-Labelling kit (PerkinElmer Inc., Turku, Finland). The antibodies were labelled at room temperature for 72 h with a 12.5-fold molar excess of the Eu^3+^-chelate in 0.1 M Na-borate buffer, pH 8.6. Excess label was removed by gel filtration on a PD 10 column (GE Healthcare Life Sciences, Uppsala, Sweden) equilibrated with 0.05 M Tris–HCl and 0.5 g/l NaN_3_ (pH 7.8). The stock conjugate was stored at 4 °C in the Tris buffer. Before use, the stock solution was further diluted to 50 μg/ml in a Tris buffer containing 75 mg/ml of diethylenetriamine pentaacetic acid-treated BSA (PerkinElmer Inc., Turku, Finland) and filtered through a 0.22-μm sterile filter (GE Healthcare Life Sciences, Germany).

### Preparation of F(ab')_2_ fragments

Monoclonal antibodies were fragmented essentially according to the method of Milenic et al. [12]. Antibody in 0.05 M Tris buffer pH 7.0 containing 0.1 M NaCl and 0.005 M EDTA was incubated at 37 °C for 2 h with bromelain (ID-Diluent 1; DiaMed AG, Switzerland) in a weight ratio of 20:1. The enzymatic reaction was stopped by the addition of 1/10 volume of freshly made 0.2 M *N*-ethyl maleimide. Products were purified at 4 °C on a protein A chromatography column equilibrated with 0.1 M sodium phosphate buffer pH 8.2 and eluted with a linear pH gradient from the pH 8.2 application buffer to a pH 3.2 limit buffer (0.025 M citric acid, 0.025 NaH_2_PO_4_, and 0.1 g/l NaN_3_). Purified F(ab')_2_ fragments were dialyzed against 0.1 M sodium borate buffer pH 8.0.

### Biotinylation of F(ab')_2_ and IgG capture antibodies

A fivefold molar excess of EZ-link Sulfo-NHS-LC Biotin (Thermo Scientific, Rockford, USA) was added to the antibodies contained in 0.15 M NaCl and 0.1 M sodium borate (pH 8.0) and incubated for 30 min at room temperature. The reaction was stopped by adding 1 M glycine, and free biotin was removed by dialysis against 0.005 M Tris–HCl, 0.15 M NaCl, and 0.5 g/l NaN_3_ (pH 7.8) or by gel filtration on a PD 10 column equilibrated with the Tris buffer. The final solution was diluted to 50 μg/ml and filtered through a 0.22-μm sterile filter.

### Epitope mapping

Epitope mapping and grouping of antibodies were performed by cross-inhibition, where binding of mAbs to the antigen was performed in the absence of competing mAbs (reference signal) or in the presence of competing mAbs. Iodinated Tg (50,000 cpm, approximately 20 ng) was allowed to react for 1 h with 1 μg competing mAbs (100 × molar excess) in PBS containing 10 g/l BSA. After incubation, 100 μl was transferred to breakapart microtiter wells coated with mAbs (1 μg/well). The plates were incubated for 1 h with shaking and then washed three times with wash solution, before counting of bound radioactivity. Binding of ^125^I-Tg without inhibiting antibody was used as a reference for each solid phase antibody, and complete cross-inhibition was defined as >80 % inhibition.

### Affinity measurements

Dissociation constants (*K*_D_) for the monoclonal antibodies were estimated from the concentration of free antibody (in mol/l) needed to achieve half-maximal binding of Tg. Tubes containing 100 μl ^125^I-Tg in 0.05 mol/l Tris–HCl with 0.1 mol/l NaCl and 0.1 % BSA were incubated with 100 μl of increasing amounts (0.32–1000 ng/tube) of the antibodies diluted in the same buffer. Free and bound antigen was separated with an excess of sheep anti-mouse antibody coupled to magnetizable polymer particles (Dynabeads M280; Life Technologies, Oslo, Norway) followed by washing and counting of radioactivity.

### Surface plasmon resonance analysis

The binding kinetics of the Tg antibodies were determined by surface plasmon resonance (SPR) using the BIAcore3000 SPR biosensor (Biacore Life Sciences, Little Chalfont, UK). Tg antibodies were immobilized at low densities (between 100 and 200 resonance units (RU)) to CM5 chips using the amino coupling protocol recommended in the BIApplications Handbook. Tg was diluted in running buffer (0.01 M Hepes pH 7.4, 0.15 M NaCl, 0.003 M EDTA, and 0.005 % Surfactant P20) to five concentration levels (0.3–25 nM) and injected sequentially with increasing concentration in a single cycle at a flow rate of 50 μl/min. The Tg association was allowed to proceed for 180 s followed by dissociation for 180 s. The temperature was kept constant at 25 °C in all runs. The reference surface, a flow cell treated with binding buffer without antigen, was used to correct for systematic noise and instrument drift in every run. Also, prior to each Tg binding cycle, buffer was injected for additional correction. The binding kinetics was calculated using the BIAevaluation software version 4.1. Association and dissociation rate constants (*k*_a_ and *k*_d_) were determined by single cycle titration using the simple biomolecular interaction model (*A* + *B* = *AB*) and corrected for mass transfer.

### Immunometric assays for thyroglobulin

Initial testing was performed with immunoradiometric assays (see Supplemental data). Further testing was performed with time-resolved immunofluorometric assays (TR-IFMAs).

#### Assay format

The TR-IFMAs for Tg were performed in streptavidin-coated 96-well microtiter plates with biotinylated mAbs on solid phase and Eu^3+^-conjugated tracer mAbs. For assaying samples, 200 μg/well of biotinylated mAb in 200 μl DELFIA assay buffer (0.05 M Tris–HCl, 0.05 M NaCl, 0.02 M diethylene triamine penta acetic acid, 0.5 g/l NaN_3_, 0.1 ml/l Tween 20, 20 mg/l Amaranth, 0.5 g/l BSA, 0.5 g/l bovine IgG, and 15 mg/l MAK33-IgG (Roche Applied Science, Penzberg, Germany), pH 7.8) was incubated under continuous shaking for 45 min before washing three times with wash solution (0.05 M Tris–HCl, 0.15 M NaCl, 0.05 % Tween 20, 0.1 % Germall, pH 7.8). Fifty microliters of calibrator or sample and 100 μl assay buffer were then added to duplicate wells, followed by continuous shaking for 60 min. Following three washes, 200 μg/well of Eu^3+^-labelled mAb in 200 μl DELFIA assay buffer was added and plates incubated for another 60 min with continuous shaking. After six washes, 200 μl/well of enhancement solution was added, followed by incubation with shaking at room temperature for 5 min. Fluorescence was measured in a time-resolved fluorometer (Victor, PerkinElmer Inc., Turku, Finland).

#### Analytical recovery

The ability of the various assays to detect Tg in the absence and presence of human anti-thyroglobulin antibodies (TgAb) was tested by pre-incubation of 180 μl of a Tg standard of approximately 18 μg/l with 20 μl of individual sera containing human TgAb ranging from 261 to 1756 kU/l or a pool of 35 different sera each containing >500 kU/l of human TgAb. The total concentration of TgAb in the pool of sera was 7225 kU/l. A sample of standard matrix buffer (0.05 M Tris–HCl, 0.15 M NaCl, 1 g/l Germall containing 60 g/l BSA, pH 7.4) replacing the TgAb fraction served as the reference.

#### Multiple antibodies combined in the assays

Antibody combinations were investigated using the IFMA procedure described above. When two or three antibodies were tested simultaneously as solid phase, 0.1 μg/well of each antibody was applied. Similarly, half of the concentration for Eu tracer was applied if two antibodies were tested in one assay. The final assay variants were performed on an automated AutoDelfia instrument.

### Calibrators and controls

The calibrators were based on purified Tg obtained from human goiter tissue, as described above. Calibrators containing 0, 0.91, 3.4, 16.6, 94.5, 442, and 1342 μg/l Tg were prepared by dilution of the purified Tg fraction in matrix buffer, and the dilutions were standardized against the certified reference material (CRM) 457 reference preparation and stored at −20 °C in aliquots. After thawing, the calibrators were stored at 4 °C and used within 2 weeks.

Control samples were PreciControls Tumor Marker from Roche Diagnostics, at two levels, 0.5 and 45 μg/l, respectively, and from pooled serum of thyroidectomized patients, with value in the low range of approximately 0.2 μg/l. The control samples were included in every run and analyzed in duplicate before and after the patient samples.

### The final TR-IFMA for Tg

The final assay for Tg was performed with two solid phase antibodies (E40 and I24) and two tracer mAbs (E44 and E45). The solid phase mAbs were modified to biotinylated (Fab')_2_ fragments, and the tracer mAbs were conjugated to the Eu^3+^-chelate, as described in earlier sections. For assaying samples, we used 100 ng/well of each biotinylated mAb and 100 ng/well of each tracer mAb, and the assay was further performed as described above.

#### Detection limit

The limit of blank (LoB) and limit of detection (LoD) were determined according to Clinical Laboratory Standards Institute (CLSI) Guideline EP17-A [13]. LoB was defined as the mean of zero calibrator +1.645 times the standard deviation (SD) and calculated from 77 replicates. LoD was defined as LoB + 1.645 times the SD of a sample containing low concentration of Tg (0.26 μg/l) and calculated from 105 replicates.

#### Functional sensitivity, precision, and linearity

The functional sensitivity was defined as the lowest Tg concentration measured with coefficient of variation (CV) of 20 %. The within-run and total imprecision of the assay were determined with 2664 samples in 60 separate runs. The concentrations of the samples were measured in duplicates using different lots of reagents and calibrators, and the testing period was 9 months. Total imprecision was defined as the CV of all measured concentrations in all assay runs and calculated using MultiCalc^TM^ software. To determine the linearity on dilution, we performed serial dilution of a serum sample with elevated Tg concentration (>1800 μg/ml). The sample was diluted 2-, 10-, 50-, 250-, 1250-, and 6250-fold in calibration buffer. The measurements were done in duplicate.

### Patient samples

Two thousand six hundred sixty-four fresh serum samples from the hospital routine laboratory were analyzed in our new highly sensitive Tg (hsTg) assay. Out of these, 1218 had Tg concentration <0.91 μg/l, 281 had Tg concentration between 0.91 and 3.4 μg/l, 553 had Tg concentration between 3.4 and 16.6 μg/l, 277 had Tg concentration between 16.6 and 94.5 μg/l, 159 had Tg concentration between 94.5 and 442 μg/l, and 176 had Tg concentration between 442 and 1342 μg/l.

In addition, 241 serum samples from 105 patients with DTC (papillary or follicular thyroid cancer) were analyzed simultaneously in our assay, a homogenous assay (Brahms Kryptor, Henningsdorf, Germany) with a measuring range between 0.09 and 200 μg/l and an electro-chemiluminescence immunoassay (Roche Diagnostics, Penzberg, Germany) with a measuring range between 0.04 and 500 μg/l. The samples were collected as part of the routine sampling procedure in follow-up after treatment of DTC in our hospital from 2009 to 2010. Seventy-four of the patients had undergone rhTSH stimulation and had low initial Tg values and negative/low values of human TgAb (<50 kU/l). We analyzed two to three samples per patient, resulting in a total of 210 samples. The final 31 samples were from 31 randomly selected patients with measurable Tg values and TgAb between 50 and 2934 kU/l. All samples were stored at −30 °C. Every participant had given written informed consent to participate in the study, and The Regional Committee for Medical Research Ethics approved the study.

### Statistical analysis

Spearman correlation and Deming regression analysis were performed to compare the new assays with the two established Tg assays from Brahms Kryptor and Roche Diagnostics. The Tg values were log-transformed to obtain normal distribution, and value <0.04 μg/l was set to 0.03 μg/l.

## Results

### Monoclonal antibody characteristics

Several monoclonal antibodies to thyroglobulin were obtained after screening approximately 2000 hybridomas from two individual fusions. From the secondary screening, nine antibodies were selected according to their ability to bind Tg in the presence of anti-thyroglobulin autoantibodies. These antibodies were subcloned and purified on a protein A column. The purified antibodies were IgG subtyped. Six antibodies (E40, E44, E47, I15, I24, and I25) were of the IgG1 subtype, while three (E39, E41, and E45) were of the IgG2_b_ subclass. All possessed kappa light chains.

### Epitope mapping

Characterization of which antibodies that was able to bind simultaneously to thyroglobulin was evaluated by cross-inhibition assays. Binding of the monoclonal antibodies to Tg was performed in the presence of inhibiting mAbs already bound to labelled antigen and in the absence of mAbs (reference signal), see Table 1. From the inhibition pattern, it was possible to classify the monoclonal antibodies into five epitope groups: A to E. Group D may be further subdivided because E39 and E44 differ in their ability to inhibit the mAb in group B.

**Table 1:** Cross-inhibition

Group	Solid phase mAb	Inhibiting antibodies								
		Group A	Group B	Group C			Group D		Group E	
		I15	I24	I25	E40	E41	E39	E44	E45	E47
A	I15	**100**	49	50	61	45	34	39	40	51
B	I24	59	**99**	47	53	35	0	23	20	41
C	I25	58	49	**99**	**98**	60	14	36	26	46
	E40	56	51	**98**	**99**	64	35	44	40	51
	E41	58	52	**90**	**95**	**98**	48	54	37	50
D	E39	57	49	63	65	66	**97**	**97**	57	64
	E44	55	48	73	74	70	**97**	**99**	38	58
E	E45	59	45	59	58	54	63	77	**99**	**99**
	E47	57	41	51	58	49	62	71	**98**	**99**

Complete inhibition >80 % is indicated in bold type

### IFMA combinations of antibody pairs

The results from the study of all antibody pairs in fluorometric assay combinations are shown in Table 2. The results indicate that antibodies belonging to groups A, B, and C are best as solid phase antibodies, while group D and E mAbs behave best as tracer antibodies. There are, however, a few other acceptable assay combinations, the other way around for solid phase and tracer mAbs from these groups. Only mAb I24 (group B) can be used both as solid phase and tracer antibody to establish an immunoassay with suitable response signals.

**Table 2:**
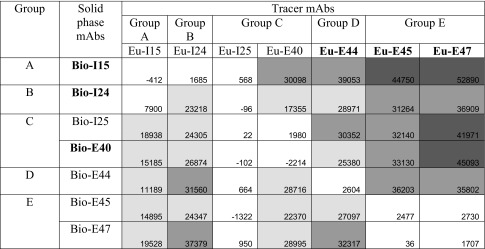
Immunofluorometric assay combinations of monoclonal antibodies

The antibodies that were further used for testing analytical recovery in the presence of interfering autoantibodies are highlighted in bold. A Tg concentration of 18 μg/ml is used in the experiment. White: cps (18 μg/ml—blank) ≤ 10,000, light grey: cps > 10,000 ≤ 30,000, grey: cps > 30,000 ≤ 40,000, dark grey: cps > 40,000

### Antibody combinations in the presence of antithyroglobulin autoantibodies

**Fig. 1:**
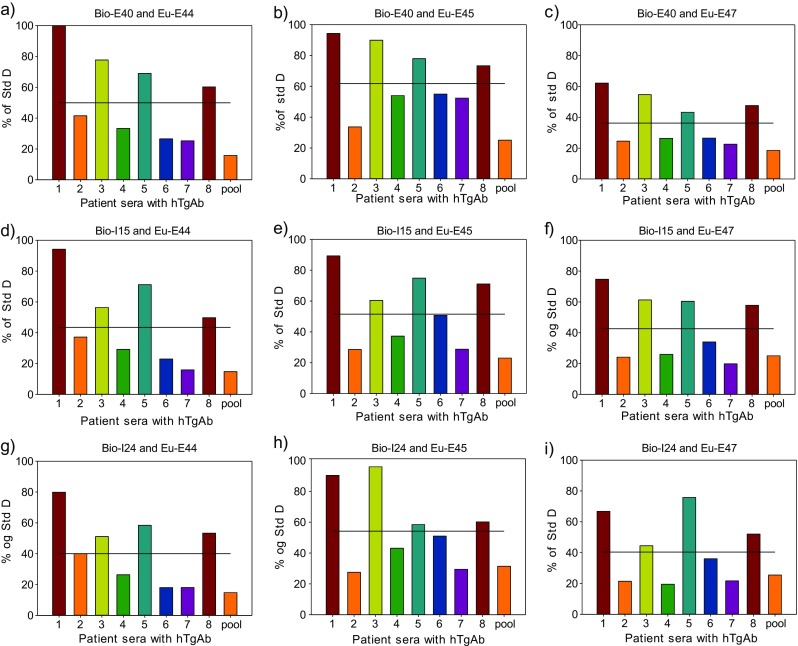
Antibody combinations in the presence of eight individual patient samples (TgAb ranging from 261 to 1756 kU/l) and one pool of patient samples (TgAb = 7224 kU/l). A Tg concentration of 18 μg/ml is used in the experiment and the results shown as percent analytical recovery. *Lines* representing mean analytical recoveries are also included in each figure

One of the purposes of this study was to select antibodies with minor influence of autoantibodies to Tg which may be present in patient serum. Thus, pairs of antibodies were tested for their ability to determine Tg in the presence of human TgAb. Initial IRMA measurements of Tg in the presence of autoantibodies indicated that mAb E40 was superior to mAb I25, both belonging to group C. Results are not shown. Therefore, the latter antibody was omitted in further experiments. Although E45 and E47 were in the same group (group E), their exceptional ability to make sensitive assays made it reasonable to include both antibodies in the further testing. Thus, we chose I15, I24, or E40 as solid phase antibodies (groups A, B, and C), respectively, with either E44, E45, or E47 as tracer antibodies (groups D and E), respectively, in the presence of a selection of patient sample containing autoantibodies. Figure 1 shows the results of these analyses. Eight individual patient sera (TgAb ranging from 261 to 1756 kU/l) and a pool of 35 sera (TgAb = 7224 kU/l) were included in IFMA combinations of the six monoclonal antibodies. Analytical recovery for the IFMA combinations differed considerably. Also, some of the individual sera were more inhibiting than others for all mAb combinations tested, and the pooled serum was highly inhibiting for all combinations tested. However, no combination was unaffected by all the autoantibodies tested. The IFMA combinations of E40 with E45 in Fig. 1b gave generally the best responses for a given concentration of Tg, while antibody combinations of E40 with E44 and I24 with E45 (Fig. 1a, h) also distinguished by the best responses in the assay for a given concentration of Tg. Thus, these results indicate that E40, I24, E44, and E45 are the best candidate antibodies for a sensitive and robust Tg assay.

### Binding kinetics of the Tg antibodies E40, E44, E45, E47, I15, and I24

The binding kinetics of the Tg antibodies were estimated using two different methods, (1) from the concentration of free antibody needed to achieve half-maximal binding of Tg and (2) by SPR analysis using the BIAcore3000 SPR biosensor (Table 3). The *K*_D_ values estimated from equilibrium analysis correlate quite well with the values obtained using SPR analysis. Both methods showed that all antibodies (E44, E45, E47, I15, and I24) had very high affinity for Tg with *K*_D_ values ≤ 6 × 10^−10^ except for E40 which had a *K*_D_ of 1 × 10^−9^. The equilibrium analysis indicated that E45 and I24 had the highest affinity for Tg with *K*_D_ = 2 × 10^−10^ for both, while the SPR analysis indicated that E47 and I15 had the highest Tg affinity with *K*_D_ = 1.1 × 10^−10^ and *K*_D_ = 2.6 × 10^−10^, respectively.

**Table 3:** Binding kinetics and affinities for the Tg antibodies E40, E44, E45, E47, I15, and I24

MAb	SPR analysis			Estimates from equilibrium analysis
	*K*_a_ [M^−1^ s^−1^]	*K*_d_ [M^−1^ s^−1^]	*K*_D_ [M]	*K*_D_ [M]
E40	3.32 × 10^5^	3.69 × 10^−4^	1.1 × 10^−9^	1.2 × 10^−9^
E44	3.14 × 10^5^	1.29 × 10^−4^	4.1 × 10^−10^	3 × 10^−10^
E45	4.99 × 10^5^	1.98 × 10^−4^	4.0 × 10^−10^	2 × 10^−10^
E47	2.95 × 10^5^	3.23 × 10^−5^	1.1 × 10^−10^	6 × 10^−10^
I15	2.46 × 10^5^	6.29 × 10^−5^	2.6 × 10^−10^	3 × 10^−10^
I24	4.24 × 10^5^	2.15 × 10^−4^	5.1 × 10^−10^	2 × 10^−10^

### IFMA combinations with multiple mAbs as catcher and tracer

Some of the immunoassays based on a pair of two antibodies were superior to others in detecting serum Tg in the presence of autoantibodies. However, none of these assays were unaffected by every analyzed sample containing human TgAb. Therefore, immunoassays combining multiple mAbs as catcher and/or tracer were evaluated. Here, we included the same six antibodies as above without excluding I15 and E47 for now. First, combinations of two or three solid phase antibodies with each of the tracer antibody candidates were tested for their performance expressed by the responses obtained in a calibration curve between 0 and 1342 μg/l. Only slight differences were seen between the calibration curves, but the combination of E40 and E24 was recognized as the best solid phase combination, results not shown. Further, combination of tracer antibodies E44 and E45 and E44 and E47, respectively, compared to only E45 were tested with the best solid phase combination (E40 + E24). Combining two tracer antibodies gave higher responses for the calibration curve compared to only one tracer, but no significantly differences were observed between the combinations E44 and E45 versus E44 and E47, results not shown. Thus, because E45 were shown to be more unaffected by human autoantibodies than E47, antibody E45 was considered a better tracer candidate than E47.

The final IFMA assay was therefore based on E40 and I24 as solid phase antibodies in combination with E44 and E45 as tracer antibodies.

### Performances of the final IFMA assay for Tg

#### Assay

**Fig. 2:**
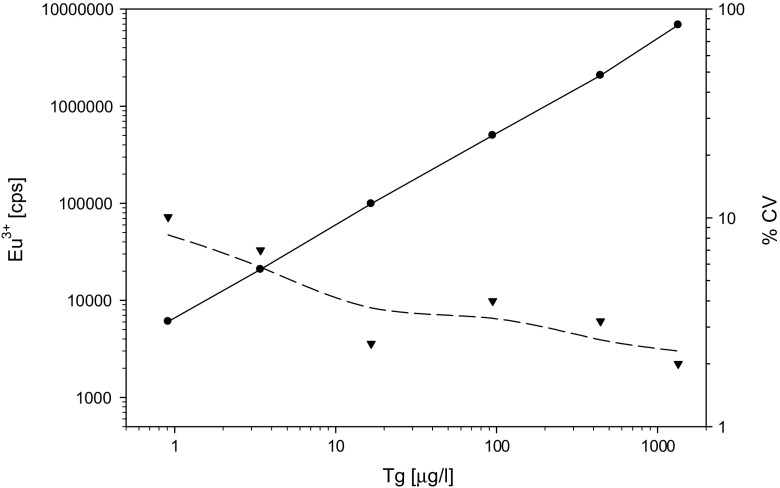
Calibration curve (*black circle*) and imprecision profile (*black down-pointing triangle*) for the final Tg assay. The calibration curve is linear over a range of 0.9–1342 μg/l (*y* = 0.96*x* + 3.81, *R* ^2^ = 1). The imprecision profile shows CV < 10 % over the entire working range (*N* = 2664)

The final assay was automated on an AutoDelfia instrument. To minimize the risk of heterophilic antibody interference, modifications were introduced including the use of F(ab')_2_ fragments as solid phase and irrelevant immunoglobulin buffer additives [14]. Patient serum diluted to 0.2, 0.45, and 42.9 μg/l Tg was used to determine optimal assay kinetics. The signals reached a plateau after a 30-min incubation for the (Fab')_2_ capture reagents, 1 h for the antigen, and a final incubation of 1 h with the Eu^3+^-labelled tracer antibodies (data not shown). A typical calibration curve is presented in Fig. 2 together with an intra-assay precision profile. The assay displayed a wide dynamic range with linearity up to 1342 μg/l.

#### Analytical validation

LoB was determined from 77 separate runs and calculated as 0.02 μg/l. LoD was determined from the LoB and SD of a sample containing low concentration of Tg (0.26 μg/l) in 105 separate runs and estimated to 0.05 μg/l. The functional sensitivity, defined as the lowest Tg concentration measured with CV of 20 %, was assessed to be 0.1 μg/l after 9 months of evaluation time using several different reagent and calibrator lots. Within-run and total imprecision of the assay were determined with 2664 samples in 60 separate runs. The variation between replicates was acceptable with a total imprecision of less than 10 % (Fig. 2). The measured mean concentrations of Tg controls were 0.26, 0.54, and 45 μg/l. The within-run imprecisions were 6.3, 4.3, and 2.3 %, and total imprecisions were 6.9, 6.9, and 3.6 %, respectively. Linearity on dilution resulted in apparent mean recoveries of 67–95 % of expected in zero calibrator.

### Comparison of the new IFMA for Tg with two well-established Tg assays

**Fig. 3:**
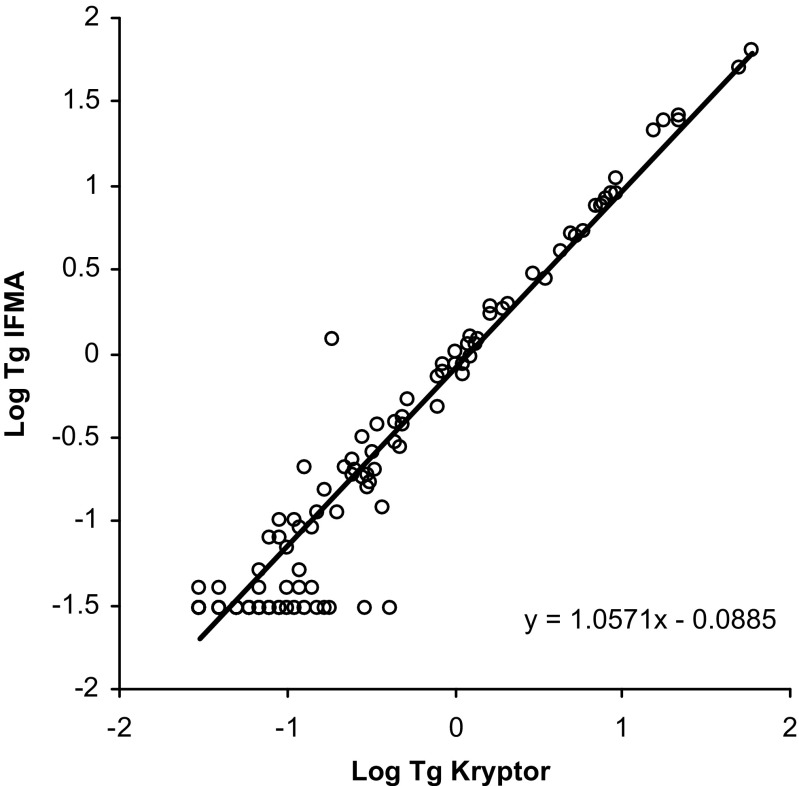
Correlation between the new TR-IFMA and a homogenous assay for detection of Tg (Brahms Kryptor) in 241 serum samples. Deming regression line is included

**Fig. 4:**
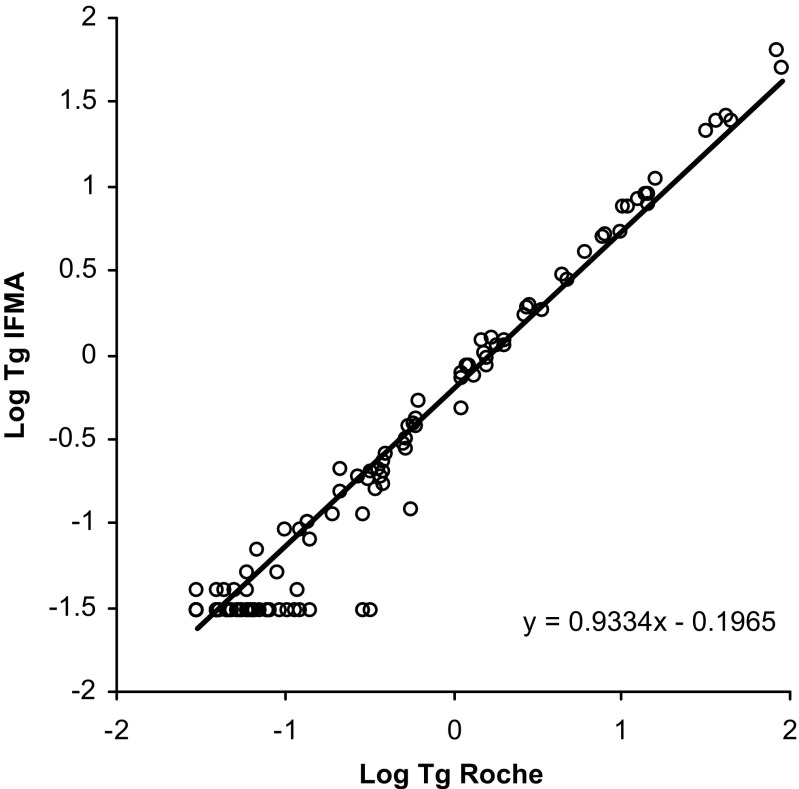
Correlation between the new TR-IFMA and a chemiluminescence assay for detection of Tg (Roche Diagnostics) in 239 serum samples. Deming regression line is included

Concentrations of Tg in 241 serum samples were determined simultaneously by the newly developed IFMA and two commercially available immunoassays for Tg, a homogenous assay (Brahms Kryptor) and a chemiluminescence assay (Roche Diagnostics). Out of these samples, 74 were from patients with low initial Tg values and negative/low values of human TgAb (<50 kU/l) and who had undergone rhTSH stimulation. We analyzed two to three samples per patients, resulting in a total of 210 samples. The final 31 samples were from 31 randomly selected patients with measurable TgAb values >50 kU/l (ranging from 50 to 2934 kU/l). The correlation between the new IFMA and the Kryptor assay was *y* = *ax* + *b* with *a* = 1.06 (95 % CI = [1.02; 1.09]) and *b* = −0.09 ((95 % CI = [−0.13; −0.04]), *ρ* = 0.79 (95 % CI = [0.74; 0.84])) (Fig. 3). Due to inadequate sample volume in two of the samples, only 239 samples were evaluated in the Roche assay. The correlation between the new IFMA and the Kryptor assay was *y* = *ax* + *b* with *a* = 0.93 (95 % CI = [0.91; 0.96]) and *b* = −0.20 ((95 % CI = [−0.23; −0.16]), *ρ* = 0.82 (95 % CI = [0.78; 0.86])) (Fig. 4). Mean difference between the new IFMA and the Kryptor assay was 0.059 μg/l (95 % CI = [−0.032; 0.15 μg/l]), and thus, the new IFMA method and the Kryptor assay gave similar results. Mean difference between the new IFMA and the Roche assay was −0.80 μg/l (95 % CI = [−1.24; −0.35 μg/l]). Thus, the new IFMA tended to give lower results than the Roche assay by between −1.24 and −0.35 μg/l.

## Discussion

Thyroglobulin is a specific tumor marker for follow-up of patients with DTC which have undergone total thyroidectomy with or without postoperative 131-iodine ablation, and the sensitivity of a Tg assay is important for a correct interpretation of clinical significance of minimal changes in serum Tg. However, the presence of anti-Tg autoantibodies may mask the epitopes used by reagent antibodies in an immunoassay and cause an underestimation of the thyroglobulin concentration. The only currently available methodology that could completely eliminate interference of TgAbs with the measurement of Tg appears to be mass spectrometry. Liquid chromatography-tandem mass spectrometry (LC-MS MS) has shown promising results for measuring Tg in biological samples with higher resistance against antibody interference than the immunoassays [8]. However, because these methods do not have the necessary functional sensitivity, have a complex and manual workflow, and are not universally available, Tg measurements with LC-MS MS should be restricted for TgAb-positive serum samples [7, 8]. Thus, there is apparently still a need for a highly sensitive Tg immunoassay with less interference by Tg autoantibodies.

To make a new and improved immunoassay for Tg, we have used a novel approach and selected monoclonal antibodies in the presence of autoantibodies from patients with thyroid cancer. After screening approximately 2000 hybridomas, nine antibodies were selected according to their ability to bind Tg in the presence of anti-Tg autoantibodies. From their cross-inhibition patterns, these antibodies were classified into five epitope groups, A to E, where group D is comprised by two subgroups. This is in agreement with earlier studies by Ruf et al. and Piechaczyk et al. where ten and 15 murine monoclonal antibodies against human Tg were classified into six epitope clusters, respectively [15, 16]. In the study by Ruf et al., two closely related groups failed to be classified in the same epitope cluster because one antibody displayed an asymmetric cross-inhibition pattern with one antibody in the other group, resembling the subdivision of our group D due to differences between mabs E39 and E44 in their ability to inhibit mAb I24 in group B. Our choice of antibodies from most of the defined epitope groups indicates that we have a representative panel of mAbs when selecting suitable antibody pairs for a new IFMA.

It is well known that some parts of the Tg molecule are more prone to autoantibody interference than other, and it has been shown that TgAbs recognize overlapping epitopes in an immunodominant region on the Tg dimer with two major and three minor epitopic regions [17]. There has also been shown that epitope recognition patterns of TgAb are different in individuals who are euthyroid or have clinical disease and that epitope recognition pattern may be of clinical and prognostic relevance in TgAb-positive DTC patients [18]. TgAbs in patients with autoimmune thyroid diseases react with a few of the antigenic determinants on Tg, and their serum levels are elevated compared to serum levels in non-autoimmune diseases [19, 20]. On the contrary, TgAbs in patients with thyroid cancer and healthy individuals appear to be more heterogeneous [19, 21, 22]. However, several studies have also been able to identify restricted TgAb recognition patterns in patients with thyroid cancer and indicated that it may be possible to distinguish between patients with different thyroid diseases and healthy individuals on the basis of the differences in TgAb specificities [20, 23]. Finally, because of the large size and structural complexity of the thyroglobulin molecule, the antigenic composition of Tg is still not well understood. Thus, it seems to be impossible to make an immunoassay for Tg completely protected against human autoantibodies. However, our approach for minimizing the influence of human TgAb was to test and select antibody pairs in the presence of human TgAb in the form of individual sera and a pool of sera consisting of 35 individual sera containing human TgAb. Consequently, some of the individual sera as well as the pooled serum were more inhibiting than others for all mAb combinations tested, and the pooled serum was most likely dominated by a few individual sera with highly elevated concentrations of human TgAb. Because some parts of the Tg molecule are more prone to autoantibody interference than other, this was as expected. In the final IFMA, we combined four antibodies against four distinct epitopes on Tg to obtain the highest sensitivity and the best possible protection against autoantibodies. Consequently, by combining several antibodies from different epitope groups, we have increased the probability of avoiding the negative effect of autoantibodies.

In the final assay, E40 and I24 were used as solid phase antibodies in combination with E44 and E45 as tracer antibodies. The antibodies were thoroughly characterized, and the binding kinetics of the antibodies was estimated from equilibrium analysis and SPR analysis. All four assay antibodies had acceptable binding affinities for construction of an immunoassay (2 × 10^−10^ < *K*_D_ <1 × 10^−9^), and the differences between the *K*_D_ values obtained with the individual methods were within expected range when using two different methods.

With a functional sensitivity of 0.1 μg/l and a total imprecision of the assay less than 10 %, our assay fulfills the criteria of a highly sensitive assay for Tg, which is useful for management of thyroid cancer [7]. As mentioned earlier, the sensitivity of the assay is important in the follow-up of DTC patients, and for the clinician, even Tg values below 0.2 μg/l may suggest no residual thyroid tissue or recurrence/metastasis of the cancer after treatment. In a study by Iervasi et al. [19], it was shown that Tg assays with sensitivities ranging between 0.1 and 1 μg/l may allow for earlier identification, particularly for patients with minimal amounts of circulating Tg [24]. It has also been shown that monitoring Tg with a sensitive immunoassay had comparable sensitivity to recombinant human TSH-stimulated Tg in follow-up of thyroid cancer patients [25]. It is likely that very low Tg concentrations do not require immediate treatment and that serial measurements of serum Tg are more informative than an absolute single value and have additional clinical benefit [26]. Thus, our new hsTg assay may have improved clinical value for detection of recurrent disease and for serial measurements in long-term follow-up.

Measuring Tg in samples with TgAb is still challenging; however, a measurable Tg value in a TgAb-positive patient gives the clinician valuable information even though the Tg value might be false low leading to further examination as ultrasonography.

Finally, there is a good agreement between the new IFMA assay and the established Tg assays from Brahms Kryptor and Roche Diagnostics; however, the new IFMA tended to give lower results than the Roche assay by between −1.24 and −0.35 μg/l. Differences in the assay antibodies characteristics may explain these discrepancies even though both assays are traceable to the same CRM 457. In a review by Clark et al., they state that although many assays are traceable to the same reference material CRM 457, a wide scatter of results was obtained [27]. The CRM 457 was shown to contain both intact Tg molecules and smaller molecular forms [27]. Thus, the different assay antibodies may differently detect these variants of the Tg molecule.

The potential underestimation of Tg concentrations caused by autoantibodies hampers the usefulness of sensitive thyroglobulin immunoassays; thus, this work can potentially improve the clinical utility of the hsTg assay.

## Electronic supplementary material

Below is the link to the electronic supplementary material.

ESM 1 (DOC 26 kb)
