# Supplementary material for: Characterization of a new highly sensitive immunometric assay for thyroglobulin with reduced interference from autoantibodies
Source: Tumour Biol. 2015 Dec 22;37(6):7729–39. doi: 10.1007/s13277-015-4597-2 (PMC4875953; doi:10.1007/s13277-015-4597-2)
Supplement: Supplementary file 1 — (DOC 26 kb) [file 13277_2015_4597_MOESM1_ESM.doc]

Supplemental data

*Antigen preparation*

Thyroglobulin was extracted from tissue taken from human thyroid goitre. The thyroid tissue was sliced and homogenized in phosphate-buffered saline, PBS, and centrifuged for 20 minutes at 10,000xg. The supernatant was subjected to fractionated ammonium sulphate precipitation. Most of the thyroglobulin was present in the solubilized precipitate after saturation between 25 and 60 % ammonium sulphate. Further separation was performed by gel filtration on a HiPrep Sephacryl-S300 column using the Akta Fast Explorer System (GE Healthcare Life Sciences, Uppsala, Sweden). This column resolved the thyroglobulin extract in three peaks, where thyroglobulin was present in the second and highest peak. Purity of combined fractions from the main peak was evaluated by SDS gel electrophoresis, which showed one main band with minor contaminations.

*Primary screening for anti-Tg monoclonal antibodies*

Primary screening was performed in Maxisorp Breakapart microtiter plates coated with 1 μg/well of sheep-anti mouse antibodies (SAM). The coating was performed as described for monoclonal antibodies. After one hour incubation with 30 µl hybridoma supernatants and 70µl PBS (0.01 M NaH_2_PO_4_, 0.15 M NaCl pH 7.2) containing 1 % BSA, the plates were washed three times with wash solution (0.05 M Tris-HCL, 0.15 M NaCl, 0.05 % Tween 20, 0.1 % Germall, pH 7.8) and 100µl ^125^I-labelled Tg (50,000 cpm/well) added followed by a second 60 minute incubation. The plates were washed three times before the wells were cut and bound ^125^I-Tg counted in a gamma counter.

*Secondary screening for anti-Tg monoclonal antibodies*

Secondary screening was performed in the presence of human anti-thyroglobulin antibodies (TgAb) obtained from patient samples. The test was performed as the primary screening except for the second incubation where the plates were incubated with 100µl ^125^I-labelled Tg (50,000 cpm/well) containing 181 kU/L of human TgAb (patient pool , 1806 kU/L). ^125^I-Tg with PBS containing 1 % BSA without TgAb served as the reference.

#### Immunoradiometric assay (IRMA) for Thyroglobulin

Maxisorp Breakapart microtiter plates coated with mAbs (1 µg/well) were incubated with 50 µl Tg (~100 µg/L) with 181 kU/L human TgAb (patient pool, 1806 kU/L) and 150 µl PBS containing 1 % BSA. ^125^I-Tg with PBS containing 1 % BSA instead TgAb served as the reference. After one hour incubation the plates were washed three times and 200 µl iodinated mAbs (50,000 cpm/well, approximately 5 ng) added. After another hour of incubation the plates were washed three times before the wells were cut and bound ^125^I-mAb counted in a gamma counter.
